# iOCT-guided simulated subretinal injections: a comparison between manual and robot-assisted techniques in an ex-vivo porcine model

**DOI:** 10.1007/s11701-023-01699-4

**Published:** 2023-09-05

**Authors:** Niklas A. Maierhofer, Anne-Marie Jablonka, Hessam Roodaki, M. Ali Nasseri, Abouzar Eslami, Julian Klaas, Chris P. Lohmann, Mathias Maier, Daniel Zapp

**Affiliations:** 1 Klinik und Poliklinik für Augenheilkunde, Technische Universität München, Ismaninger Str. 22, 81675 Munich, Germany; 2 Translational Research Lab, Carl Zeiss Meditec AG, Munich, Germany; 3 Klinik und Poliklinik für Augenheilkunde, Ludwig-Maximilians-Universität München, Munich, Germany

**Keywords:** Robotic eye surgery, Robotic injection, Vitreoretinal surgery, Subretinal injection, Minimally invasive ophthalmic surgery, Robotic surgery, Ophthalmology

## Abstract

The purpose of this study is to compare robot-assisted and manual subretinal injections in terms of successful subretinal blistering, reflux incidences and damage of the retinal pigment epithelium (RPE). Subretinal injection was simulated on 84 ex-vivo porcine eyes with half of the interventions being carried out manually and the other half by controlling a custom-built robot in a master–slave fashion. After pars plana vitrectomy (PPV), the retinal target spot was determined under a LUMERA 700 microscope with microscope-integrated intraoperative optical coherence tomography (iOCT) RESCAN 700 (Carl Zeiss Meditec, Germany). For injection, a 1 ml syringe filled with perfluorocarbon liquid (PFCL) was tipped with a 40-gauge metal cannula (Incyto Co., Ltd., South Korea). In one set of trials, the needle was attached to the robot’s end joint and maneuvered robotically to the retinal target site. In another set of trials, approaching the retina was performed manually. Intraretinal cannula-tip depth was monitored continuously via iOCT. At sufficient depth, PFCL was injected into the subretinal space. iOCT images and fundus video recordings were used to evaluate the surgical outcome. Robotic injections showed more often successful subretinal blistering (73.7% vs. 61.8%, *p* > 0.05) and a significantly lower incidence of reflux (23.7% vs. 58.8%, *p* < 0.01). Although larger tip depths were achieved in successful manual trials, RPE penetration occurred in 10.5% of robotic but in 26.5% of manual cases (*p* > 0.05). In conclusion, significantly less reflux incidences were achieved with the use of a robot. Furthermore, RPE penetrations occurred less and successful blistering more frequently when performing robotic surgery.

## Introduction

Numerous retinal pathologies, such as inherited RPE dystrophies or age-related macular degeneration (AMD), share similar terminal stages characterized by a decline of the retinal pigment epithelium (RPE). Physiologically, the functions of RPE include (1) nutrition of photoreceptor cells, (2) phagocytosis and recycling of outer receptor segments, (3) absorption of scattered light, (4) regulation of retinal fluids and—(5) as part of the blood–retinal barrier—the preservation of the intraocular immune privilege [1–3]. RPE disorders are, therefore, associated with a disturbed retinal homeostasis leading to loss of photoreceptor cells and vision.

In general, three different therapeutical approaches based on subretinal injection have been established in retinal research: while the injection of tissue plasminogen activator (tPA) close to AMD-associated submacular hemorrhages has already found its way into clinical practice [4, 5], subretinal gene therapy and stem cell injections are the subjects of current animal experimental and clinical studies. In 2017, Voretigen Neparvovec (Luxturna®) was approved by the US Food and Drug Administration as the first subretinally administered gene therapy for the treatment of RPE65 mutation-associated retinitis pigmentosa and Leber congenital amaurosis [6, 7]. Packaged into recombinant adeno-associated virus (AAV) or lentivirus vectors, gene transfer is applied via subretinal injection as close to RPE as possible, while intravitreal vector injections have turned out to be less effective, most likely due to impermeability of the internal limiting membrane [8–10].

Considering the retina’s fine anatomy with an average thickness of approximately 250 µm [11, 12] on the one hand and the surgeon’s physiological cannula-tip tremor in the order of 100–200 µm [11–13] on the other hand, surgical drug delivery into the target structure proves to be a challenging task. Furthermore, poor visual depth information increases the risk for a sub-RPE located or suprachoroidal drug administration. The use of microscope-integrated intraoperative optical coherence tomography (iOCT) for tracking the cannula tip relative to RPE has already been described in various papers approaching subretinal injection [3, 10, 11, 14]. However, the injection procedure itself still demands a high degree of precision and remains a main surgical concern. It is, therefore, important to consider alternative drug delivery strategies into the subretinal space.

Requirements for successful subretinal injections are low injection pressures to prevent retinal layer ruptures [15, 16] as well as small tip diameters [17] and tissue-conserving mechanical movements to keep retinotomy tight and minimize reflux [18]. This study aims to examine the feasibility of meeting these requirements using a remote-controlled telemanipulation device. Therefore, manual and robot-assisted subretinal injections were performed in 84 ex-vivo porcine eyes after standard pars plana vitrectomy (PPV) and compared in terms of successful subretinal blistering, occurrence of reflux and RPE damage. A surgical microscope with integrated OCT was used for both manual and robot-assisted procedures. Robot-assisted trials were performed with a custom-built telemanipulation device that was controlled by the surgeon using a 3D mouse.

The porcine model was chosen for its quick post-mortem availability and considered a sufficient approximation of the human anatomy [19–21], as the occurrence and measurement of events determining failure and success could be replicated reliably. Furthermore, since this study is an evaluation of a proof-of-concept, no human donor resources were wasted.

For simulating subretinal drug deposition, perfluorocarbon liquid (C_8_F_18,_ PFCL) was chosen. PFCL to a large degree remains in the local area of application and does not distribute considerably within the subretinal space. This way, its delivery could easily be evaluated post-operatively on intraoperatively captured OCT data. In the case of reflux, intravitreal PFCL was also clearly visible as homogeneous foreign matter inside the vitreous cavity allowing the assessment of further complications.

## Methods

Eighty-four (*n* = 84) ex-vivo porcine eyes obtained from healthy rearing pigs (aged 5–7 months) were operated on by two specially trained investigators (STI) either manually or robot-assisted, comprising a total of 21 surgeries per surgeon in each setting. The STI with no prior experience in vitreoretinal surgery underwent a comprehensive wetlab training guided and surveilled by a more than 20 years experienced vitreoretinal surgeon. In addition, the STI were introduced into handling and adjustment of the microscope and iOCT, both in terms of hardware and software. One subretinal injection was performed per eye. The order of trials was randomized interpersonally and intermethodically. During subretinal injection, the non-operating STI was acting as an assistant continuously tracking the cannula in cross and longitudinal section with OCT. In addition to optical fundus video recording, real-time OCT snapshots and 3D-cube captures of size 3 × 3 mm and 10 × 10 mm were taken prior to penetration of the retina, after cannula-tip positioning just above RPE and after subretinal injection.

### Preparation and instruments

Immediately after slaughter and enucleation at a local abattoir, porcine eyes were transported to the laboratory on ice and immersed in cooled balanced salt solution (BSS) for a maximum of 4 h before interventions [21–23]. Only optically clear eyes were selected for surgery and fixated on a rubber base imitating the orbital cavity. For this purpose, two pins were placed in the temporal and nasal conjunctive tissue enabling passive eye movements. Lubricating eye drops (Vidisic EDO 0.6 ml; Bausch & Lomb/Mann Pharma, Berlin, Germany) were used to moisten the cornea during the surgical procedure.

Sclerotomy took place via three Geuder AG made single-use trocars of size 23-gauge. Cutter (25-gauge), light pipe, external air compressor and foot pedal controlling cutting frequency and aspiration were connected to VISALIS V500 phaco and vitrectomy system (Carl Zeiss Meditec AG, Jena, Germany). Avoiding air pockets, PFCL (7 ml; Fluoron GmbH, Ulm, Germany) was aspired into a disposable 1 ml syringe using a 27-gauge cannula. The syringe was then tipped with a metal bent micro-needle (40-gauge, diameter 80 μm) carried by a 23-gauge stem cannula (Incyto Co., Ltd., South Korea).

### Microscope and intraoperative OCT

A standard ophthalmic surgery microscope equipped with an OCT engine (OPMI LUMERA 700 with RESCAN 700, Carl Zeiss Meditec AG, Jena, Germany) was used for all experiments. Two OCT B-scans crossing one another perpendicularly were continuously captured by the microscope at a desired location controlled by the assistant using the auxiliary screen. The operating field was directly observed through the ocular of the microscope with the two B-scans and an OCT acquisition location marker virtually projected onto the scene.

### Robot

**Fig. 1:**
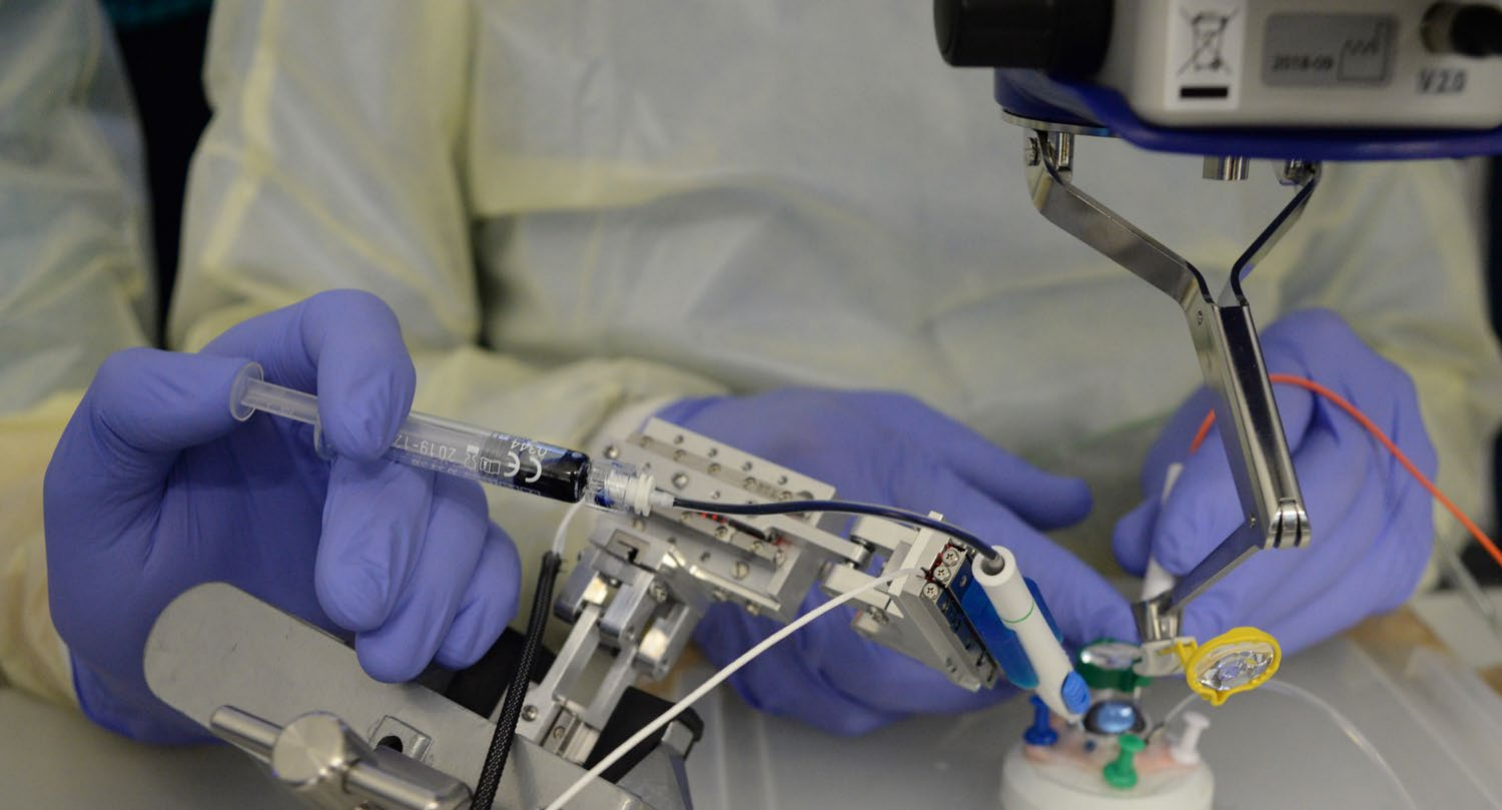
The custom-made robot equipped with injection cannula and Heidelberger extension. After the surgeon has maneuvered the cannula tip to the retinal target site using a 3D mouse, the assistant injects PFCL via a syringe attached to the Heidelberger extension

For robotic trials, a custom-made hybrid parallel-serial robot presented in [24, 25] was used, whose joints comprised prismatic piezo actuators (Fig. 1). The robot had five degrees of freedom and was remote-controlled in a master–slave fashion by operating a 3D mouse. Collective joint activation enabled precisely coordinated movements of the syringe connected to the robot’s end joint. To avoid unwanted bulb movements during the injection procedure, the instrument trocar was set as cannula fulcrum point (remote center of motion, RCM) when inserting the needle tip into the eye. All robotic movements were remote controlled by the STI without the use of automatisms or deep learning techniques.

### Surgical technique

**Fig. 2:**
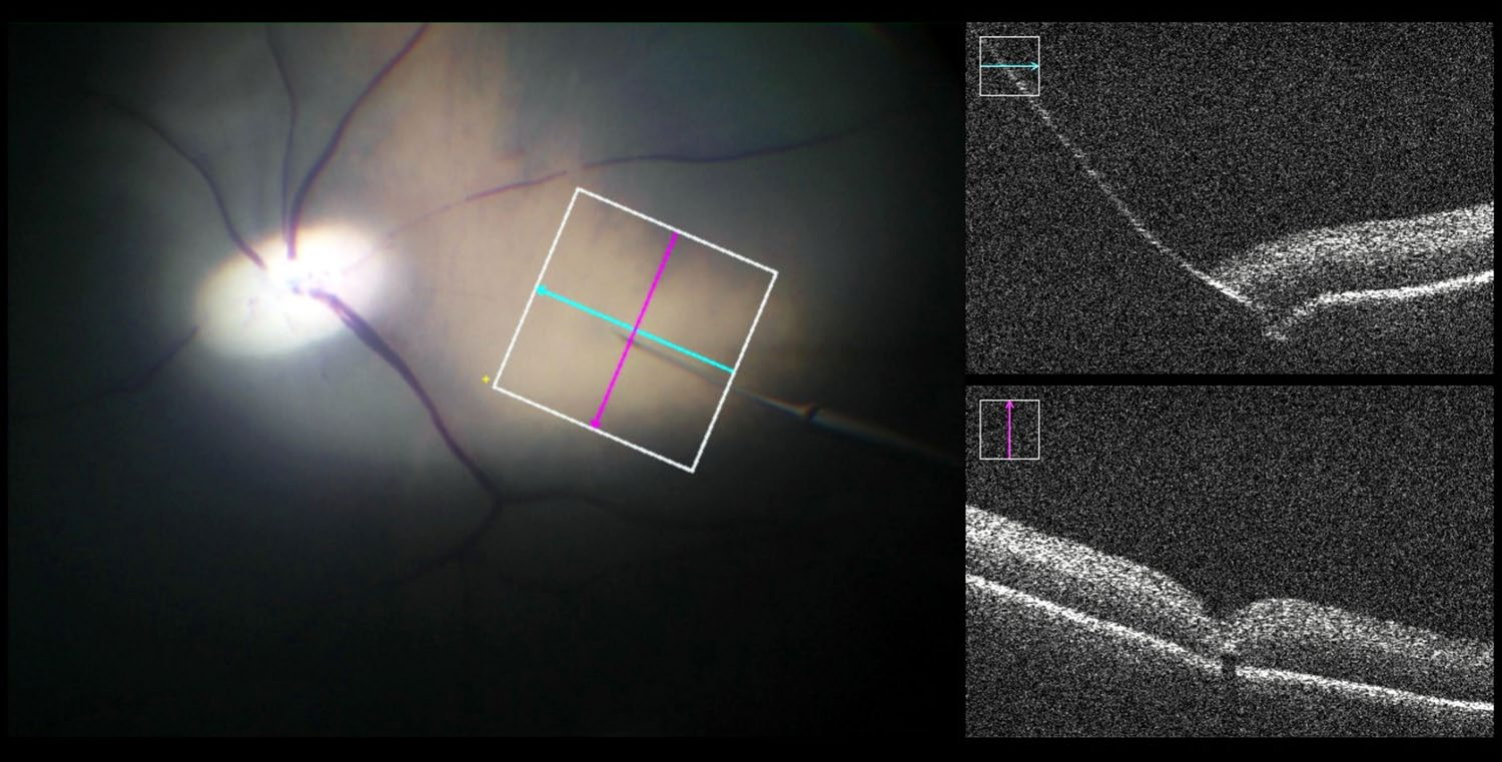
Microscopic view (left) and intraoperative OCT acquisitions (right) from a robot-assisted trial immediately before subretinal injection of PFCL. The cyan and magenta arrows indicate OCT capture locations and show the cannula’s longitudinal (top right) and cross section (bottom right). The cannula tip is placed close above RPE while avoiding its penetration

**Fig. 3:**
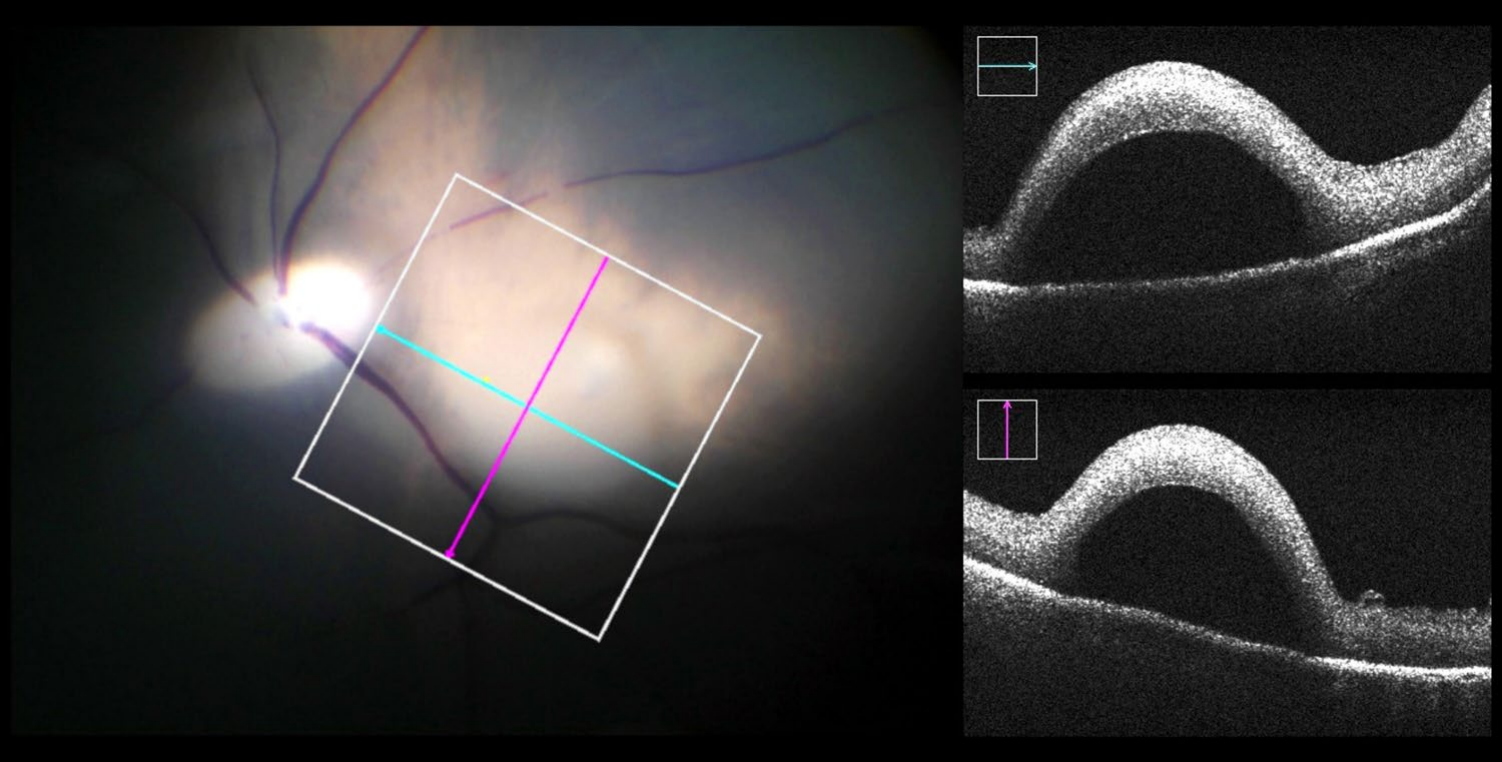
An example of a successful subretinal blistering visible in microscopic view (left) and intraoperative OCT acquisitions (right). Leaving RPE undamaged in its initial position, the subretinally discharged PFCL causes a detachment of retinal layers. Visually, the subretinal bleb appears as a dull protrusion of the fundus. The cyan and magenta arrows indicate OCT capture locations

For sclerotomy, three 23-gauge trocars were placed at approximately 60°, 120°, and 240° with a distance of 3 mm to the limbus. While maintaining intraocular pressure with BSS irrigation, pars plana vitrectomy was performed manually using a 25-gauge posterior vitrectomy probe. Subsequently, the retinal target site for injection was specified by the assistant STI. The desired injection area was mapped on iOCT using the vertical cross formed by OCT longitudinal and cross section. Depending upon the surgical method, the PFCL-filled syringe was either attached to the robot’s end member or used by hand. In robot-assisted trials, the tip of the injection cannula was approached to the trocar manually by moving the robot unit as a whole. The final insertion of the tip into the trocar, which also served as RCM, was enabled using translational robotic movements in *x*-, *y*-, and *z*-directions, with the cannula direction being defined as *z*-axis and the plane perpendicular to it as xy plane. The retinal target area was then approached at an angle of 30° to 60° relative to the retinal surface and carefully penetrated by gradually advancing along *z*-axis only under iOCT guidance (Fig. 2). Once the cannula tip was as close as possible to RPE without touching it, PFCL was slowly injected into the subretinal space until retinal detachment and blistering appeared in OCT (Fig. 3). As Kwon et al. report, an initial pressure of 25 to 35 psi is necessary to induce retinal lifting. However, further injection was aimed at with lower pressures to reduce retinal stretching and minimize the risk of reflux into the vitreous [16].

### Parameters

For statistical comparison between manual and robot-assisted surgery, the following variables were analyzed: surgical outcome, occurence of reflux, retinal damage/RPE penetration, cannula penetration depth presented in terms of retinal surface to RPE percentage, cannula angle relative to RPE, intraretinal tunnel length and duration of procedure. A subretinal injection was considered successful if all of the following conditions are met: (1) subretinal blistering above RPE, (2) no observed RPE penetration or other signs of retinal damage and (3) no bleb rupture when retracting the cannula. The occurrence of reflux was rated as failure if no subretinal bleb formation was observed. All variables were assessed and calculated post-operatively in a synopsis of microscopic video recordings and geometrically corrected OCT B-scans (fan-beam method).

### Statistical analysis

Data collected on the study are reported as mean ± standard deviation. Differences between groups were calculated using two-tailed Chi-square test for categorial and independent two-tailed Student’s *t* test for continuous variables. *P* values below 0.05 (*p* < 0.05) were considered statistically significant. Binary logistic regression was used to compare the two STIs’ success and reflux rates. For learning curve analysis, power function trendlines were fitted to the charts showing durations over attempts. Learning percentages were then estimated using power law of learning. All statistical analyses were conducted using SPSS version 1.0.0.1461 (SPSS Inc., Chicago, IL, USA).

## Results

Out of 84 available pig eyes, 72 were included in the study. 12 eyes were excluded due to cloudy corneas and poor imaging quality (8), pre-existing retinal detachment (3) or as a result of damage to the needle tip when inserting the cannula into the trocar (1).

The robot-assisted procedure led to a successful blistering in 73.7% of the cases compared to 61.8% with the manual method. Out of ten unsuccessful robotic attempts, 50% were due to no or minimal blistering caused by reflux, 40% to RPE damage and 10% to the rupture of a subretinal bleb when retracting the cannula. The 13 unsuccessful attempts at manual intervention were also due to RPE damage (69.2%), lack of blistering (23.1%) and bleb rupture (7.7%). With the present case number of 72, however, success rates did not achieve any statistical significance (*p* = 0.279). Success and failure rates can be obtained from Table 1.

**Table 1:** Statistical comparison between manual and robot-assisted surgery

		Manual		Robotic		Significance (two-tailed)
		Success (61.8%)	Failure (38.2%)	Success (73.7%)	Failure (26.3%)	
Occurrence of reflux (percentage)		58.8%		23.7%		Yes (*p* < 0.01)
Occurrence of RPE damage (percentage)		26.5%		10.5%		No (*p* = 0.079)
Cannula entry angle (degrees)	Mean	39.2°	39.1°	44.6°	49.0°	
	STD	14.1°	8.0°	11.4°	8.0°	
Achieved tip depth (retinal percentage)	Mean	87.2%		83.1%		No (*p* = 0.107)
	STD	5.7%		7.9%		
Cannula tunnel length (micrometers)	Mean	427.9 μm		355.5 μm		No (*p* = 0.144)
	STD	124.7 μm		149.4 μm		
Operation duration (seconds)	Mean	32 s		120 s		Yes (*p* < 0.001)
	STD	17 s		65 s		

Means and standard deviations (STD) are given for continuous variables

**Fig. 4:**
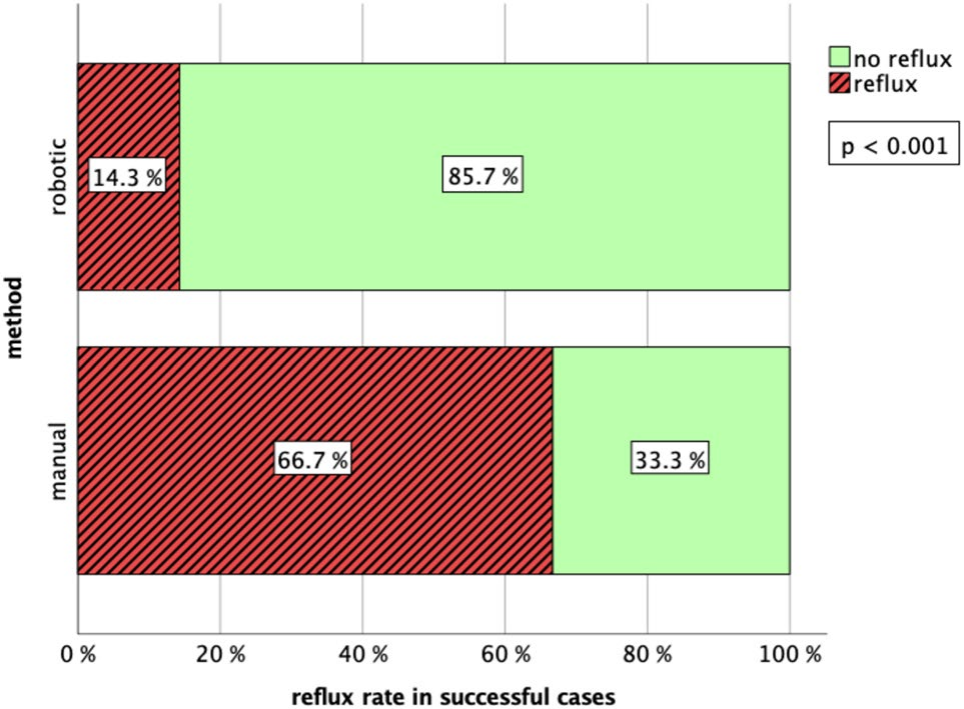
Bar diagram showing reflux rates in manual and robot-assisted trials that featured successful subretinal blistering. At 14.3%, robotic surgery shows significantly (*p* < 0.001) less reflux than surgeries carried out manually (66.7%)

A significant difference between the two methods was evident regarding the occurrence of intravitreal reflux. 58.8% of manual and 23.7% of robot-assisted trials were associated with reflux (Table 1, *p* < 0.01). This difference became even clearer if only successful subretinal injections were considered (Fig. 4). When using the robot, reflux occurred in 14.3% of successful cases compared to 66.7% with manual surgery (*p* < 0.001). In both manual and robotic successful trials, an average volume of 0.008 ml ± 0.004 ml of PFCL was injected.

**Fig. 5:**
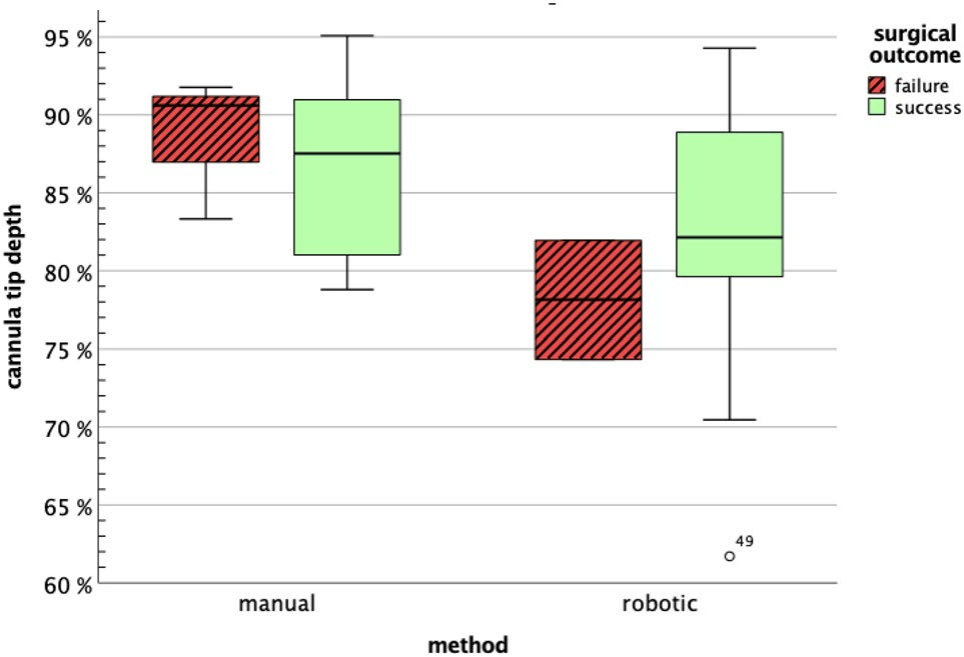
Box plot showing the achieved tip depths in manual and robot-assisted trials for which no penetration of the RPE was observed. The inner band represents the median. Injections with successful blistering (green boxes) are found at depths of approximately 80–90% in both methods, with manual trials achieving slightly larger depths on average

When approaching the RPE along *z*-axis, the achieved tip depth was set in relation to the distance between retinal surface and RPE. Considering only successful trials without penetrating RPE, a larger depth was achieved manually (87.2% ± 5.7%) compared to robotic trials (83.1% ± 7.9%) (Fig. 5). However, less RPE penetration was observed when using the robot (Table 1). Damage to the RPE was found in 10.5% of all robotic and 26.5% of all manual cases. As summarized in Table 1, neither the differences in depth nor penetration rate showed statistical significance.

The mean intraretinal tunnel length was 427.9 μm ± 124.7 μm in successful manual and 355.5 μm ± 149.4 μm in successful robot-assisted trials, and the mean cannula entry angle relative to RPE was 39.2° and 44.6° (Table 1).

The average duration of a successful surgery from the moment of entering the trocar until cannula retraction was 120 s ± 65 s in robot-assisted and 32 s ± 17 s in manually performed cases. Considering the durations of successful injections over time, no learning curve effect was found for the robot-assisted setting. In the case of manually performed trials, a learning percentage of 83.05% was observed. Between the two STIs, no notable differences in terms of method, success, and reflux rate could be found.

## Discussion

From this series of experiments, two main conclusions can be drawn. First, the combination of robotic assistance and intraoperative OCT guidance was more likely to achieve successful blistering than manual OCT-guided injection. Less harming of RPE in robot-assisted trials must, to a large extent, be attributed to better OCT visualization of the cannula-tip location within the retina. In manual trials, visualization was largely affected by hand tremor, as the needle tip could not remain steady at a certain depth; robotically, however, a more precise estimation of the needle tip location was possible throughout the steady advancement of the tip. Neither the observed differences in the success rate nor those in the occurrence of RPE penetration showed statistical significance, probably due to an insufficient case number. For cannula-tip depth analysis, the maximum relative tip depth achieved at any point of a successful insertion procedure was considered. As shown in Table 1, larger penetration depths were observed in manually performed trials. A possible explanation might also be tremor: when inserting the needle and injecting PFCL, manual trials showed slight tip fluctuations along the cannula axis, since the retinal tissue hardly presents any haptic resistance. As a result, this lack of control over slight maneuvers led to unwanted more-than-aimed penetration depths. Although deep insertions are desirable and, thus, manual attempts appear to be more successful in achieving that, their uncontrollable nature makes manual surgeries less secure. This observation is also supported by the fact that penetrations of the RPE did, indeed, occur more frequently in the manual setting compared to robot-assisted trials.

Second, in robot-assisted surgery, post-injection reflux has been shown to occur significantly less often. A potential explanation could be based on the dynamic of the cannula during injection and retraction: in manual trials, the cannula tip did not remain in a stable position during injection. During retraction, linear retraction along negative *z*-axis without oscillations were largely impossible. As a result, the retinal puncture was subject to forces following *x*- and *y*-axes. Due to the enlargement of the intraretinal tunnel, leakage increased and thus permitted the applied fluid to escape the subretinal space. Robot-assisted retraction, however, showed fewer oscillations following *x*- and *y*-axes and followingly likely had less impact on the size of the puncture. As mentioned above, effectiveness of substances intended for subretinal injection must be questioned if spread into the vitreous cavity by reflux [8–10]. A reduction of the amount of reflux observed can, therefore, be considered an important criterion for cost-efficiency of future therapeutic applications involving costly pharmaceutical agents. Followingly, the rarer occurrence of intravitreal reflux when using the robot can be regarded as quality indicator for the subretinal injection procedure.

On the other hand, it was found that performing robotic injections takes significantly more time than performing manual injections. A major reason for this might be the more precise and therefore also slower but more controlled movements observed in robotic trials. The influence of precision versus time efficiency needs further discussion in future work. Suggestions are made for improving the robotic setup to reduce preparation and operation time. Furthermore, considering the durations of successful injections over time, we have observed that STIs tend to learn manually performed injections with an 83% learning percentage and less with robotically performed ones. Since the risk in robotic subretinal injection is not known, a risk-adjusted cumulative sum analysis is not possible and normal cumulative sum analysis would not provide further information compared to our learning analysis. Since this study was not designed to capture learning, there are insufficient data for a more detailed analysis.

The off-label usage of PFCL simulating the application fluid has proven to be considerably beneficial for investigating the injection procedure: PFCL did, to a certain degree, remain in the area of subretinal deposit. Provided that it has been applied successfully, PFCL in intraoperative OCT was displayed as a subretinal bleb, allowing for the analysis of the continuity of RPE and the retinal layers located above the subretinal space. In the event of reflux back into the vitreous, PFCL visually appeared as a stationary, homogenous sphere leading to the step formation in OCT (due to changed refractive index) while only slightly impairing the microscopic view into the eye. This allowed for the detection of even small amounts of reflux. Furthermore, the lack of intravitreal diffusion also enabled the assessment of the retinal integrity further away from the puncture site. Obstruction of the 40-gauge cannula has not been observed. No problems occurred in dosing the applied volume. For the purpose of this study, the use of heavy liquid was, therefore, a good approximation of the drug delivery process. As a hydrophobic substance, however, PFCL and its fluid dynamic properties cannot be considered entirely representative, since gene vectors and pharmaceuticals are often in a hydrophilic form.

Besides the usage of PFCL, several limitations regarding the validity of this study must be considered. Most importantly, the use of ex-vivo porcine eyes was suboptimal. To assess robotic feasibility with subretinal injection procedures, it is essential to replicate the above-described experiments with human donor eyes. Although measurement and analysis of success and complication parameters have been possible in this ex-vivo porcine model, it is important to consider that retinal properties may differ from human eyes. Effects on the parameters examined cannot be excluded. It is also important to mention differences between ex-vivo and in-vivo eyes, such as the retina’s adhesion to the RPE. The findings of this study were gathered from investigations on STIs without prior experience in human vitreoretinal surgery. This work can, therefore, primarily be regarded as a proof-of-concept. Further research with experienced surgeons, both on human donor eyes and in an in-vivo setting, is required. However, robotic assistance in combination with OCT guidance presents an interesting approach in training surgeons.

In summary, it has been demonstrated that the use of a robotic telemanipulation device is feasible and advantageous when performing subretinal injection. Only when combining depth information provided by iOCT and stable robotic motion sequences, continuous cannula-tip tracking was enabled. In our opinion, this not only reduces trauma to the surrounding retinal tissue, but also surgeons in particular benefit from real-time visual feedback. Further applications in posterior chamber surgery are conceivable and need to be investigated. It will also be necessary to investigate to what extent robotic interventions can benefit from storable movement patterns and deep learning techniques. When injecting viral vectors into the subretinal space, for example, a previous injection of BSS has proven to be useful. On the one hand, this serves to ensure successful retinal bleb formation and, thus, in case of failure, not to waste expensive therapeutic agents [26, 27]; on the other hand, retinal overstretching and dilution can be reduced [27]. Using the robot, it would be possible to precisely reproduce the first injection trajectory for the second insertion, which means that BSS and therapeutic agent could be applied via the same retinotomy. It has to be examined whether tissue trauma and leakage can be further minimized this way.

## Data Availability

The data used to support the findings of this study can be viewed on request.
